# Analysis of Sex, Age and Disease Factors Contributing to Prolonged Life Expectancy at Birth, in Cases of Malignant Neoplasms in Japan

**DOI:** 10.2188/jea.13.169

**Published:** 2007-11-30

**Authors:** Tomoyuki Watanabe, Masako Omori, Hiromi Fukuda, Hiroki Takada, Masaru Miyao, Yutaka Mizuno, Isao Ohsawa, Yuzo Sato, Toshihiko Hasegawa

**Affiliations:** 1 Graduate School of Medicine, Nagoya University.; 2 Graduate School of Science, Nagoya University.; 3 Information Technology Center, Nagoya University.; 4 Obu Dementia Care Research and Training Center.; 5 Research Center of Health, Physical Fitness and Sports, Nagoya University.; 6 National Institute of Public Health.

**Keywords:** life expectancy, malignant neoplasms, mortality

## Abstract

BACKGROUND: This study aimed to examine the contribution made by the change in mortality from malignant neoplasms to the life expectancy at birth, observed during the years 1965-1995 in Japan.

METHODS: We used data on the population and number of deaths by cause, age and sex in 1965, 1975, 1985 and 1995. The contribution of different ages and causes of death to the change in life expectancy were examined with the method developed by Pollard.

RESULTS: We found that, among all causes, the decrease of mortality from stomach cancer led to the greatest improvement in life expectancy for both sexes. On the other hand, negative contributions were seen with cancers of many sites, such as cancer of the intestine, liver and lung for males, and cancer of the intestine, gallbladder, lung and breast for females. Recently, the contributing years of all cancers have been negative because of the increase in mortality from malignant neoplasms. In addition, increase of death from malignant neoplasms in middle-aged and elderly people negatively influenced the life expectancy at birth.

CONCLUSIONS: Female cancer influenced the improvement in life expectancy at birth. Cancer for males, however, contributed little to improvement of life expectancy at birth except for a little prolongation of life expectancy at birth during the years 1965-1975. To develop a public health policy, the contributing years to life expectancy at birth can be a useful indication in evaluating the impact of death from various diseases. It is necessary to analyze the contribution made by various causes of death to the changes of life expectancy at birth.

In Japan, the leading causes of death have changed dramatically over the past several decades, from infectious diseases such as tuberculosis in earlier years to cancer, cerebrovascular disease and cardiovascular diseases today. As a result of greatly overcoming various diseases such as infectious diseases and cerebrovascular and cardiovascular diseases, life expectancy at birth among Japanese has been the longest in the world since 1984, and longer than at any time in history.^1^ However, malignant neoplasms have been the leading cause of death among Japanese since 1981 and continue to show upward trends today, despite various measures against cancer. In 2000, the number of deaths from malignant neoplasms among Japanese was 295 484, accounting for about 30% of the total.^2^ Cerebrovascular disease had long been the leading cause of death until it began decreasing greatly in the latter 1960s. In 1981, it was overtaken by malignant neoplasm and became the second leading cause of death. Since then, it has continued to decline, and today ranks third among causes of death. Heart disease, formerly third among the causes of death, became the number two cause of death in 1985. It showed a temporary decline in recent years, but has now regained its former high position.^2^ Compared with other countries, a greater decline in cancer mortality over the last few years has been reported in the European Union.^3^^,^^4^ A similar decline in cancer mortality has been observed in the United States of America,^5^^-^^7^ but cancer mortality among Japanese has moved upward.

Life expectancy at birth among Japanese continues to increase, despite the upward trend in mortality rates from malignant neoplasms seen in Japan today. In other words, the change in mortality from malignant neoplasms is not a factor in the improvement of life expectancy at birth. Malignant neoplasms may, however, prevent the prolongation of life expectancy at birth. To evaluate the influence of each disease on the change of life expectancy at birth, several studies have investigated and analyzed the contributions to changes in life expectancy at birth by cause of death and age group.^8^^-^^10^ Although there have been almost no such studies in Japan, we reported the contributions of change in circulatory disease mortality to the life expectancy at birth among Japanese, observed during the years 1955-1995.^11^

In this study, we examined the contribution of different age groups and causes of death, especially malignant neoplasms, to the change in life expectancy at birth.

## METHODS

To study the contribution of change in mortality from malignant neoplasms to the life expectancy at birth by age, we classified the data into 5 groups according to subject age as follows: 0-14, 15-44, 45-64, 65-74, and 75 years or over. The basic data used in the present study to analyze the contributions in these 5 groups were population size and the number of deaths by cause, age (5-year groups), and sex in 1965, 1975, 1985, and 1995 from the vital statistics of Japan.^12^^-^^14^ Life expectancies were estimated from the complete life tables in 1965, 1975, 1985, and 1995.^1^ We analyzed the changes in cause of death for each of the following 10-year periods: 1965-1975, 1975-1985, and 1985-1995. All the diseases examined were cancers; namely, cancers of the stomach; small intestine including the duodenum and colon (intestine); rectum, rectosigmoid junction and anus (rectum); liver and intrahepatic bile ducts (liver); gallbladder and extrahepatic bile ducts (gallbladder); trachea, bronchus and lung (lung); breast; uterus; prostate and other sites. The causes of death were classified according to the International Classification of Diseases and Causes of Death (ICD) as shown in Table 1.

**Table 1.** Classification of causes of death by malignant neoplasms

	Calendar year (ICD version)			
	
Site of malignant neoplasm	1965	1975	1985	1995
	(ICD-7)	(ICD-8)	(ICD-9)	(ICD-10)
Stomach	151	151	151	C16
Small intestine, including duodenum and colon	152 and 153	152 and 153	152 and 153	C17 and C18
Rectum, rectosigmoid junction and anus	154	154	154	C19-C21
Liver and intrahepatic bile ducts	155.0, 155.8, and 156	155, 197.7, and 197.8	155 and 199.1c	C22
Gallbladder and extrahepatic bile ducts	155.1	156	156	C23 and C24
Trachea, bronchus and lung	162 and 163	162	162	C33 and C34
Breast	170	174	174 and 175	C50
Uterus	171-174	180-182	179-182	C51-C55, and C58
Prostate	177	185	185	C61

When a life table was applied at the two time points t_1_ and t_2_, the difference in life expectancy at birth e_0_^2^-e_0_^1^ depended on the changes in mortality for each age group at the two time points. Thus, using the method of Pollard,^15^^-^^17^ we were able to evaluate the differences in life expectancy at birth at these two times produced by changes in mortality from different causes of death. The method of Pollard is based on the concept that the difference in life expectancy at birth between two time points can be calculated by summing the difference of appropriately weighted mortality by cause and age at the two points of time.

Thus, we weighted changes in life expectancy against changes in mortality by malignant neoplasms, and calculated this as contributing years.

The following approximate formula can be used to analyze change in life expectancy at birth according to mortality trends by age and cause:

$$\begin{array}{rl} e_{0}{}^{2}-e_{0}{}^{1} & ≅\sum_{\text{i}}{(}_{1}m_{0}{}^{\text{(i)1}}-{}_{1}m_{0}{}^{\text{(i)2}})w_{0}+4\sum_{\text{i}}{(}_{4}m_{1}{}^{\text{(i)1}}-{}_{4}m_{1}{}^{\text{(i)2}})w_{2}\\ & \;+5\sum_{\text{i}}{(}_{5}m_{5}{}^{\text{(i)1}}-{}_{5}m_{5}{}^{\text{(i)2}})w_{7.5}+5\sum_{\text{i}}{(}_{5}m_{10}{}^{\text{(i)1}}-{}_{5}m_{10}{}^{\text{(i)2}})w_{12.5}+\cdots \\ \;\text{with} & \;w_{\text{t}}=1/2{(}_{\text{t}}p_{0}{}^{2}e_{\text{t}}{}^{1}+{}_{\text{t}}p_{0}{}^{1}e_{\text{t}}{}^{2}),\;{}_{\text{n}}m_{\text{x}}{}^{\text{(i)}}={}_{\text{n}}m_{\text{x}}{(}_{\text{n}}D_{\text{x}}{}^{\text{(i)}}{/}_{\text{n}}D_{\text{x}}) \end{array}$$

where the suffixes 1 and 2 on the life table functions and mortality rate refer to times 1 and 2; _t_p_x_^1^, _t_p_x_^2^ is the probability of survival t years from x at times 1 and 2, respectively; e_x_^1^, e_x_^2^ is the expectation of life at age x; and _n_m_x_^(i)1^, _n_m_x_^(i)2^ is the central mortality rate for cause i in an age interval (x, x+n-1) at times 1 and 2, respectively. _n_D_x_ is the number of deaths from all causes in an age interval (x, x+n-1), and _n_D_x_^(i)^ is the number of deaths by cause i in an age interval (x, x+n-1). Finally, w_t_ is the weight function.

We could calculate life expectancy at birth by integrating the force of mortality (or instantaneous death rate) at age x. By dividing the age range [0, ∞) into the short intervals [0,1), [1,5), [5,10), [10,15) ···(where “[a,b)” represents “a≦x<b”), this integral was approximated for each short interval. We could calculate the contributing years by cause, using the central mortality rate weighted by the proportion of the number of deaths from malignant neoplasms against the number of deaths from all causes.

A positive contribution indicates that a mortality reduction in the relevant age group contributes to an increase in life expectancy at birth, whereas a negative contribution indicates that a mortality increase contributes to a reduction in life expectancy at birth. For this paper, we estimated the contributions of malignant neoplasms to changes in life expectancy by age group and cause of death.

## RESULTS

Table 2 shows the contributing years for age and sites of malignant neoplasms from 1965 to 1995 for males and females, respectively. The contributing years of all causes to the changes of life expectancy at birth were 3.931 years (1965-75), 3.057 years (1975-85) and 1.587 years (1985-95) in males and 3.913 years (1965-75), 3.614 years (1975-85) and 2.346 years (1985-95) in females. The total improvement of life expectancy at birth showed downward trends in both sexes. The contribution of malignant neoplasms accounted for 1.8% (1965-75), -2.7% (1975-85) and -2.3% (1985-95) in males and 5.2% (1965-75), 5.8% (1975-85) and 6.6% (1985-95) in females. Therefore, the contributions of malignant neoplasms to the changes of life expectancy at birth were small during the years 1965-1995.

**Table 2.** Contributions of changes in mortality from malignant neoplasms to life expectancy at birth between 1965 and 1995

Age (year)	Male			Female		
	
	1965-1975	1975-1985	1985-1995	1965-1975	1975-1985	1985-1995
All causes						
0-14	0.944	0.587	0.146	0.786	0.457	0.120
15-44	0.582	0.491	0.239	0.546	0.353	0.133
45-64	1.085	0.573	0.423	0.950	0.708	0.304
65-74	0.809	0.810	0.253	0.834	0.860	0.483
75-	0.511	0.596	0.526	0.797	1.236	1.306
Total	3.931	3.057	1.587	3.913	3.614	2.346

All cancers						
0-14	0.006	0.017	0.011	0.004	0.012	0.011
15-44	0.013	0.040	0.041	0.047	0.058	0.049
45-64	0.088	-0.040	0.060	0.142	0.138	0.053
65-74	0.001	0.009	-0.082	0.033	0.065	0.058
75-	-0.038	-0.109	-0.068	-0.023	-0.062	-0.017
Total (% ^# 1^)	0.070 (1.8)	-0.083 (-2.7)	-0.037 (-2.3)	0.202 (5.2)	0.211 (5.8)	0.155 (6.6)

Stomach						
0-14	0.000	0.000	0.000	0.000	0.000	0.000
15-44	0.019	0.032	0.023	0.017	0.034	0.035
45-64	0.140	0.128	0.099	0.086	0.098	0.068
65-74	0.062	0.103	0.058	0.049	0.077	0.054
75-	0.001	0.023	0.035	0.005	0.026	0.047
Total (%)	0.221 (5.6)	0.287 (9.4)	0.215 (13.6)	0.157 (4.0)	0.236 (6.5)	0.204 (8.7)

Intestine ^# 2^						
0-14	0.000	0.000	0.000	0.000	0.000	0.000
15-44	-0.003	0.001	0.001	-0.005	0.000	0.002
45-64	-0.010	-0.019	-0.013	-0.008	-0.015	-0.009
65-74	-0.006	-0.012	-0.021	-0.006	-0.009	-0.007
75-	-0.005	-0.012	-0.016	-0.005	-0.014	-0.018
Total (%)	-0.023 (-0.6)	-0.042 (-1.4)	-0.049 (-3.1)	-0.023 (-0.6)	-0.038 (-1.0)	-0.031 (-1.3)

Rectum ^# 3^						
0-14	0.000	0.000	0.000	0.000	0.000	0.000
15-44	-0.004	0.004	0.004	-0.002	0.005	0.002
45-64	-0.003	-0.010	-0.010	-0.002	0.004	0.002
65-74	-0.003	0.001	-0.007	0.000	0.006	0.003
75-	-0.002	-0.002	0.002	-0.003	0.003	0.003
Total (%)	-0.012 (-0.3)	-0.008 (-0.2)	-0.011 (-0.7)	-0.007 (-0.2)	0.017 (0.5)	0.010 (0.4)

Liver ^# 4^						
0-14	0.000	0.000	0.000	0.000	0.000	0.001
15-44	-0.001	-0.001	0.005	0.003	0.003	0.003
45-64	-0.007	-0.093	-0.007	0.017	0.003	-0.002
65-74	-0.001	-0.018	-0.055	0.010	0.003	-0.021
75-	0.002	-0.009	-0.013	0.003	-0.002	-0.009
Total (%)	-0.008 (-0.2)	-0.121 (-4.0)	-0.069 (-4.4)	0.033 (-0.8)	0.007 (0.2)	-0.030 (-1.3)

Gallbladder ^# 5^						
0-14	0.000	0.000	0.000	0.000	0.000	0.000
15-44	0.000	-0.001	0.001	-0.001	-0.001	0.002
45-64	-0.004	-0.004	0.000	-0.010	-0.006	0.012
65-74	-0.005	-0.010	-0.001	-0.013	-0.012	0.010
75-	-0.003	-0.012	-0.007	-0.007	-0.022	-0.005
Total (%)	-0.013(-0.3)	-0.027(-0.9)	-0.007(-0.5)	-0.031(-0.8)	-0.040(-1.1)	0.019(0.8)

Lung ^# 6^						
0-14	0.000	0.000	0.000	0.000	0.000	0.000
15-44	-0.001	-0.004	0.000	0.001	-0.001	-0.001
45-64	-0.023	-0.027	-0.001	-0.006	-0.005	-0.008
65-74	-0.038	-0.041	-0.029	-0.010	-0.012	0.003
75-	-0.018	-0.054	-0.034	-0.007	-0.025	-0.017
Total (%)	-0.080(-2.0)	-0.126(-4.1)	-0.064(-4.0)	-0.022 (-0.6)	-0.043 (-1.2)	-0.024 (-1.0)

Breast						
0-14	0.000	0.000	0.000	0.000	0.000	0.000
15-44	0.000	0.000	0.000	-0.005	-0.006	-0.007
45-64	0.000	0.000	0.000	-0.016	-0.011	-0.033
65-74	0.000	0.000	0.000	-0.002	-0.005	-0.006
75-	0.000	0.000	0.000	-0.001	-0.001	-0.005
Total (%)	0.000 (0.0)	0.000 (0.0)	0.000 (0.0)	-0.025 (-0.6)	-0.023 (-0.6)	-0.050 (-2.1)

Uterus						
0-14	-	-	-	0.000	0.000	0.000
15-44	-	-	-	0.028	0.009	-0.001
45-64	-	-	-	0.062	0.059	0.016
65-74	-	-	-	0.012	0.026	0.014
75-	-	-	-	0.002	0.005	0.007
Total (%)	-	-	-	0.105 (2.7)	0.099 (2.8)	0.036 (1.5)

Prostate						
0-14	0.000	0.000	0.000	-	-	-
15-44	0.000	0.000	0.000	-	-	-
45-64	-0.001	0.000	-0.003	-	-	-
65-74	-0.002	-0.003	-0.008	-	-	-
75-	-0.003	-0.010	-0.012	-	-	-
Total (%)	-0.005(-0.1)	-0.012(-0.4)	-0.023(-1.4)	-	-	-

Other sites						
0-14	0.005	0.017	0.011	0.003	0.012	0.011
15-44	-0.003	-0.015	-0.022	-0.033	-0.032	-0.023
45-64	-0.086	0.043	-0.053	-0.120	-0.116	-0.034
65-74	-0.002	-0.003	0.073	-0.037	-0.063	-0.038
75-	0.035	0.093	0.056	0.016	0.042	0.007
Total (%)	-0.075(-1.9)	0.066(2.2)	0.020(1.2)	-0.184 (-4.7)	-0.204 (-5.6)	-0.122 (-5.2)

Values and percentages in this column may not add up exactly to the total number and 100% because of rounding, respectively.

In this study, we excluded the sites of cancer for which the contribution from changes in malignant neoplasm mortality against changes in life expectancy from all causes of death was small (under about 1%) during each of the 10 years.

^# 1^ The proportion of the contribution from changes in malignant neoplasm mortality against changes in life expectancy from all causes of death.

^# 2^ Intestine: Small intestine, including duodenum and colon

^# 3^ Rectum: Rectum, rectosigmoid junction and anus

^# 4^ Liver: Liver and intrahepatic bile ducts

^# 5^ Gallbladder: Gallbladder and extrahepatic bile ducts

^# 6^ Lung: Trachea, bronchus and lung

The contributions of death from all cancers to the change in life expectancy at birth between 1965 and 1975 were 0.070 years for males and 0.202 years for females. In terms of the sites of malignant neoplasms, the contributing years for stomach cancer was the largest for males (0.221 years) and females (0.157 years). The major causes showing a positive contribution were uterine cancer (0.105 years) and liver cancer (0.033 years) for females. On the other hand, male lung cancer (-0.080 years) and female gallbladder cancer (-0.031 years) had the largest negative contribution. Other large negative contributions were due to intestinal cancer (-0.023 years) for males, and cancer of the breast (-0.025 years) and lung (-0.022 years) for females.

By age group, a negative contribution was seen among the middle-aged and elderly in both sexes. The majority of the positive contribution was accounted for by stomach cancer in age 45-64 (males: 0.140 years, females: 0.086 years). Death from male lung cancer in age 65-74 and female breast cancer in age 45-64 was the major factor shortening life expectancy, -0.038 years and -0.016 years, respectively.

The contribution of total cancer to the changes of life expectancy at birth between 1975 and 1985 was -0.083 years among males and 0.211 years among females. Cancer in males showed a negative contribution during this decade. Stomach cancer in both sexes showed the largest weight in improvements in life expectancy (0.287 years and 0.236 years, males and females, respectively), as is the case with the years 1965-75. Female uterine cancer (0.099 years) and rectal cancer (0.017 years) were the major causes contributing positively. A negative contribution with the largest weight was seen in lung cancer (-0.126 years), liver cancer (-0.121 years) and intestinal cancer (-0.042 years) for males and cancer of lung (-0.043 years), gallbladder (-0.040 years) and intestine (-0.038 years) for females.

By age, stomach cancer at ages 45-64 in both sexes accounted for the majority of the positive contribution to the changes of life expectancy at birth (males: 0.128 years, females: 0.098 years). Conversely, the major factors for reduced life expectancy at birth were liver cancer in the 45-64 age group for males (-0.093 years) and lung cancer in the 75 years and older age group for females (-0.025 years).

The number of years contributed by age group during the years from 1985 to 1995 showed a decrease for males (-0.037 years) and increase for females (0.155 years). The major cause of death showing a positive contribution was stomach cancer in both sexes (males: 0.215 years, females: 0.204 years). Other causes contributing positively were uterine cancer (0.036 years) and cancer of the gallbladder (0.019 years) in females. The leading causes showing reduction of life expectancy at birth were liver cancer (-0.069 years) in men and breast cancer (-0.050 years) in women. In addition, lung cancer (-0.064 years) and intestinal cancer (-0.049 years) for males and cancer of the intestine (-0.031 years) and liver (-0.030 years) for females contributed negatively to the changes of life expectancy at birth.

By age, reduction of mortality from stomach cancer among the 45-64 age group made the most positive contribution in both sexes (males: 0.099 years, females: 0.068 years). Conversely, the major causes and age groups showing a negative contribution were liver cancer in the 65-74 age group for males (-0.055 years) and breast cancer in the 45-64 age group for females (-0.033 years).

## DISCUSSION

In this study, we evaluated the contribution of the change in mortality from malignant neoplasms to the life expectancy at birth during the years 1965-1995 in Japan. The decreased mortality from stomach cancer for both sexes had a great impact on the improvement in life expectancy. On the other hand, factors showing a negative contribution by increasing mortality were cancer of the intestine, liver and lung for males, and cancer of the intestine, gallbladder, lung and breast for females. Negative contributions were seen for cancers of many sites in both sexes. In recent years, the contributing years of all cancers has been negative because of an increase in mortality from malignant neoplasms. As a result of an analysis similar to ours, Ngongo et al. reported the negative contribution of lung cancer for both sexes and breast cancer for females to the changes of life expectancy at birth in Italy during 1980-1992.^10^ Their results showed the same trends as in our study.

Stomach cancer contributed positively to the changes of life expectancy at birth in both sexes among all age groups. As the contributing years at age 45-64 was the highest of all age groups, the mortality from stomach cancer in middle age was decreased. Many studies have reported a continuing decline in mortality from stomach cancer in various countries including Japan.^18^^-^^21^ However, deaths from stomach cancer account for the majority of cancer deaths recently. The number of deaths from stomach cancer in young and middle aged groups has decreased, but the number of deaths in elderly groups remains high. In fact, Levi reported that Japanese mortality from stomach cancer at age 65-84 years was the highest in the industrialized countries in the early 1990s.^22^

The contribution of cancer of the intestine among Japanese has been negative for both sexes. The contributing years at age 45 years or over were negative. Intestine cancer at age 45-64 showed the most negative contributions during the years 1965-1985 in both sexes. However, the age groups showing the most negative contributions shifted to age 65-74 for males and 75 years or over for females during the years 1965-1985. In Russia, intestinal cancer mortality showed an upward trend as in Japan.^23^ Conversely, colon cancer mortality in the USA has showed a downward trend recently.^24^

Lung cancer contributed negatively to the changes of life expectancy at birth in those aged 15 years or over in both sexes. The negative contribution was the highest at age 65-74 during the years 1965-1975 and at age 75 years or over during the years 1975-1995. In Europe as in Japan, mortality from lung cancer has showed an upward trend for both sexes over the last 40 years. Lung cancer mortality at age 35-64 years, however, showed a downward trend for both sexes in Spain, Italy and the United Kingdom, contrary to Japan.^21^ In the USA, lung cancer mortality for females showed an upward trend, while male lung cancer mortality showed a downward trend recently.^24^

Male liver cancer contributed to the largest reduction of life expectancy at birth at age 45-64 during the years 1965-1985 and at age 65-74 during the years 1985-1995. Female liver cancer during the years 1965-1985 contributed to an improved life expectancy at birth among almost all age groups while liver cancer for females showed a negative impact at age 45 years or over during the years 1965-1985. In Japan, liver cancer mortality for both sexes began to increase in the late 1970s.^25^ Afterwards mortality from liver cancer showed an upward trend for males and was generally flat for females. In the last 20 years, mortality from hepatocellular carcinoma has shown upward trends in Japan, the UK, France and the USA.^26^^-^^30^

The changes in mortality from uterine cancer among almost all age groups contributed to the large improvement of life expectancy at birth during the years 1965-1995. Especially, uterine cancer showed the largest contribution at 45-64 age groups. One of the reasons was that uterine cancer mortality among Japanese showed a continuous downward trend, as in Europe and the USA.^21^^,^^31^^,^^32^

The contribution of cancer of the breast at age 15 years or over has been negative during the years 1965-1995. The negative contributing years were the largest in the 45-64 age group. Breast cancer mortality at age 20-69 years declined by 22% in the UK and 19% in the USA between 1981 and 1997.^33^ In the European Union as a whole, the overall age-adjusted mortality rate from breast cancer showed a downward trend in recent years.^34^^,^^35^An increase of mortality from breast cancer among Japanese, however, was a factor reducing life expectancy at birth.

Using the method of Pollard, we could evaluate the extent to which changes of mortality in two different time periods influenced the changes in life expectancy at birth by cause and age group. Moreover, this method provides a more accurate estimate of the influence on contributing years by weighting deaths occurring at younger ages more heavily than those occurring in older age groups.^15^^-^^17^ In this study, we found that the positive or negative contribution of malignant neoplasms to the changes of life expectancy at birth was much larger in elderly people than in young people. This is the reason mortality from malignant neoplasms among elderly groups produced remarkable positive or negative changes of life expectancy at birth, while cancer mortality among younger age groups has little impact on changes of life expectancy.

Improvement of measures to overcome cancer such as stomach cancer screening and treatment lead to an increased life expectancy at birth in young and middle aged people under 64 years old. However, the contribution of cancer has been negative for males 65 years or over and female 75 years or over, and the proportion of aged people dying from cancer has increased. One method to estimate the contributions by cause of death is known as the potential gains in life expectancy by complete elimination of a specific cause of death.^36^^,^^37^ The potential gains are regarded as the expectation of life lost by death from cancer. This means that the impact of death from cancer on life expectancy becomes larger as potential gains increase. The potential gain in life expectancy at birth (life expectancy at age 0) by elimination of deaths from cancer ranked second behind that from elimination of cerebrovascular disease in 1965 (males: 2.02 years, females: 1.98 years). Since then, it has continued to increase remarkably, and ranked first in 1995 (males: 3.84 years, females: 2.76 years). The potential gains in life expectancy at age 65 by elimination of deaths from cancer were estimated at 1.07 years for males and 2.69 years for females in 1965 against 2.69 years and 1.72 years for males and females in 1995, respectively.^1^ Compared with the potential gains in life expectancy at birth by elimination of deaths from cancer, the potential gains in life expectancy at age 65 were large. It is suggested that a high proportion of aged people died from cancer.

In conclusion, female cancer influenced the improvement in life expectancy at birth. Cancer in males, however, contributed little to improvement of life expectancy at birth except for a little prolongation of life expectancy at birth during the years 1965-1975. Quantitatively evaluating the burden of diseases in a population and computing the potential impact on deaths from various diseases such as cancer is important in the development of a public health policy. Therefore, the contributing years to life expectancy at birth can be a useful indicator in evaluating the impact of death from various diseases. In the future, it will be necessary to analyze the contribution to the changes of life expectancy at birth by various causes of death.
